# Evaluation of the factors affecting triage decision-making among emergency department nurses and emergency medical technicians in Iran: a study based on Benner’s theory

**DOI:** 10.1186/s12873-022-00729-y

**Published:** 2022-10-28

**Authors:** Aghil Habibi Soola, Saeid Mehri, Islam Azizpour

**Affiliations:** 1 Department of nursing, School of Nursing and Midwifery, Ardabil University of Medical Sciences, Ardabil, Iran; 2 Department of Emergency nursing, School of Nursing and Midwifery, Ardabil University of Medical Sciences, Ardabil, Iran

**Keywords:** Triage decision-making, Emergency Department Nurses, Emergency Medical Technicians, Benner’s theory, Iran

## Abstract

**Introduction:**

Emergency department (ED) nurses and emergency medical technicians (EMTs) find themselves performing triage under time pressure and with limited information. Identifying an effective triage decision-making process can play a significant role in promoting patient safety. Experts are able to make faster and more effective decisions in emergencies than novices.

**Objective:**

The current study aimed to identify the level of triage decision-making (TDM) and its’ predictors in ED nurses and EMTs based on self-reported levels of nursing proficiency in Benner’s theory from novice to expert.

**Materials and methods:**

Out of 821 ED nurses and EMTs who met the inclusion criteria, 320 ED nurses and 152 EMTs were included in this descriptive-analytical research. Data were collected by a demographic information form and triage decision-making inventory (TDMI) and analyzed by SPSSv.22 software using descriptive statistics, Pearson correlation test, t-test, ANOVA, and multiple linear regression.

**Results:**

The total score of TDMI in the ED nurses and EMTs was higher in the expert nurses than in the proficient, competent, advanced beginner and novices. Multiple linear regression analysis showed that self-reported levels of nursing proficiency, age, work experience, marital status and triage training course were predictors of TDM in ED nurses (P < .05), and self-reported levels of nursing proficiency, service location, work experience, and triage training course were predictors of TDM in EMTs (P < .05).

**Conclusion:**

Understanding the predictors influencing TDM health professionals may facilitate the understanding of their training needs. The training needs of a novice and inexperienced person may be different from those of an expert person, it is recommended that the training methods be based on the experiences and professional levels of nurses so that the training provided is effective and quality. Moreover, to increase the TDM power and reduce TDM errors due to lack of experience, a system is suggested to be established to allow novice nurses in the first year to work with experienced nurses. Also it is suggested that the determining educational and training focus with regards to triage before entering the bedside be done based on predictors.

## Introduction

Triage is an essential part of emergency medical care that refers to the process of prioritizing patients to receive health care in the emergency department (ED) and emergency medical services (EMS) without wasting time [1, 2]. It is an independent nursing role that involves critical thinking and clinical decision-making skills [3]. ED nurses and emergency medical technicians (EMTs) find themselves performing triage under time pressure and with limited information [4, 5]. Because the accuracy and speed of triage assessment have been linked to patient safety outcomes, nurses must be accountable for their decisions [3]. In prioritizing a patient’s urgency for an accurate category and triage decision-making, the presence of expert and skilful emergency nurses is essential to determine the best effect of the intervention on the patient’s health [6]. Experts are able to make faster and more effective decisions in emergencies than novices [5].

The history of EMS dates back to 1975, when the first emergency department was established in Tehran. After that, pre-hospital emergency departments were gradually formed in other provinces of Iran. These parts consist of urban centers and road centers [4, 7]. EMS staff in Iran have an associate’s or a bachelor’s degree in emergency medicine or a bachelor’s degree in nursing [4, 8]. Two staff members must be present in each shift [8]. In the pre-hospital setting, emergency medical technicians (EMTs) face a wide range of time constraints, environmental hazards, lack of diagnostic facilities, and work-related stress. Therefore identifying high-risk patients in the pre-hospital setting is important. In EMS, patients are mostly triaged based on experience and Gestalt impressions of EMTs [1, 9]. The purpose of pre-hospital triage is to find high-risk patients at the scene and treat them as soon as possible. Triage is done based on the answers to three questions, including is there a need to transfer the patient to medical centers? Which hospital is more suitable for the patient according to the required distance and specialization? And what are the most appropriate means to transport the patient? [10, 11]. In many EDs, EMS-reported assessments and vital signs play an integral role in ED triage. In Iran, however, emergency nurses rely more on their initial assessments and re-evaluate all patients regardless of the reports of EMS providers [1].

In the ED, the primary goal of triage is to identify patients in need of emergency intervention. The secondary goal is to ensure that patients receive safe treatment within a specified time frame [6]. Triage in ED is standardized in the shortest possible time based on factors such as the patient’s vital signs, physical examination, level of consciousness, mechanism of trauma, and underlying conditions such as age and comorbidities. In this type of triage, the patient is examined for about one minute, and at the end, they are divided into three levels (immediate, emergency, and delayed) or five levels (critical, immediate, emergency, relatively urgent, and delayed) [10]. In Iran, EDs use the five-level emergency service index (ESI) due to their simplicity, easy training, perceptual and comprehensive approach, and operational nature [1].

Triage decision-making (TDM) is presented as a clinical decision-making skill in the triage evaluation phase, including prioritizing care and deciding on the interventions that will follow [12]. TDM is an important part of emergency care because it categorizes patients based on their severity to manage patient flow. Triage decisions are often made quickly, independently, and under time constraints and can have a great impact on patient outcomes and flow [13]. Early identification and prioritization of patient problems and prioritization of nursing care are essential skills for nurses working in different clinical settings around the world [14]. On the one hand, correct decision-making can lead to cost reduction, proper use of human resources, and improved quality of care. Further, it creates a professional identity in nurses and differentiates professional nurses from non-professional care personnel [15]. Factors influencing triage decisions include the use of intuition, clinical experience, and cognitive factors [16, 17]. Clinical experience is associated with increased reliability and accuracy of TDM among nurses. Experienced nurses have a range of experiences when judging possible situations. Experience and expertise have been shown to be effective factors in decision-making at different levels of nursing [18]. In their 2013 study, Smith et al. mention that the triage decision-making skills of novice nurses are completed by gaining clinical experience [14]. The difference between the performance of novice and expert nurses in triage decision-making is essential to understanding their strengths and weaknesses [5].

Benner (1982) provided a theoretical framework for the novice to expert model. This model discusses how a person starts at the beginner stage, and through the acquisition of new skills and knowledge, progresses through several stages to end at the specialist level. The five skill levels in this model are novice, advanced beginner, competent, proficient, and expert [19]. The way information is interpreted and discussed varies between novices and experts. The difference in decision-making based on proficiency is that novice and advanced beginners do not consider different options and competent ones make a conscious assessment, while expert nurses make decisions based on their experiences so that there is no need for conscious analysis [20, 21]. Expert nurses and appropriate triage decisions reduce waiting time and improve patient management, while unskilled personnel and incorrect triage decisions lead to waste of resources, delays in patient treatment, dissatisfaction, and adverse consequences [4, 6].

Success in ED and EMS depends on several factors, one of the most important of which is the ability to make good clinical decisions [4]. Therefore, it is necessary to identify and address those challenges to improve the quality of emergency services. A broad search of the literature showed no study that assessed, compared, and evaluated the effective predictors of TDM in ED nurses and EMTs based on self-reported levels of nursing proficiency in Benner’s theory from novice to expert. On the other hand, having such information is important and necessary to decide on interventions that improve the quality of TDM. Because the outcome of a triage decision has implications for patient safety and health resource utilization [9], this study was aimed to investigate level of TDM and identify its predictors in the ED nurses and EMTs based on their reported nursing skill levels according to Benner’s theory.

## Methods

### Study design

The statistical population of this descriptive-analytical study consisted of all ED nurses and EMS staff in Ardabil province. Ardabil University of Medical Sciences consists of all EDs and EMS services and has 52 EMS centers and 10 hospital emergency departments. The inclusion criteria for the study consisted of working for a minimum of six months in the ED/EMS and being active during the data collection stage. All those on break during the study time and incomplete questionnaires were excluded from the research. The convenience sampling method was adopted to select the samples. Initially, the researchers obtained a permit from the Ethics Committee of the University of Medical Sciences and received a letter of recommendation from the Vice Chancellor for Research. The letter was presented to the officials of EMS centers and educational hospitals in Ardabil province. Then, the researchers referred to 10 teaching hospitals and all EMS centers in Ardabil province. They were introduced to the emergency nurses and EMS by the nursing offices of the mentioned centers and EMS officials. Before sampling, a short introduction of the study design and purpose was presented to the prospective participants. The paper version of the questionnaire was then distributed among the participants by the researchers. Tis descriptive-analytical study was performed from March to April 2021 in Ardabil province, north-western Iran. Finally out of 821 nurses who met the inclusion criteria, 218 did not consent to participate in the study, 89 questionnaires were not returned, and 42 questionnaires were incomplete. Finally, 320 ED nurses and 152 EMTs were included in the study by completing a questionnaire.

### Data collection tools

Participants completed a demographic data questionnaire which included age, gender, work experience, work shift, marital status, level of education, triage training course, service location, and self-reported levels of nursing proficiency from novice to expert based on Benner’s theory.

Triage decision-making inventory (TDMI) was developed by Cone [22] with good validity and reliability to examine the TDM skills among nurses and was used in other studies 6, 12, 13, 23]. The original questionnaire consisted of 37 items and 4 subscales, which was reduced to 27 items with three subscales. In a subsequent study, the original factors of Critical Thinking and Cognitive Characteristics were merged into one factor relabeled as Cognitive Abilities. The 27-item questionnaire applies to nurses working in all types of clinical settings. The questionnaire includes cognitive abilities (14 questions), experience (6 questions), and intuition (7 questions). The questionnaire is scored based on a 6-point Likert scale (1 = strongly disagree to 6 = strongly agree). The total summative score is 162 for the 27 items [16]. The whole score of this questionnaire and its subscales is obtained by dividing the sum of the scores given by the participants by the total number of questions. Cone used the mean total score for TDMI as a cut-off point [23]. In the main study, Cronbach’s alpha ranged from 0.85 to 0.92 for each subscale [16]. In our study, Cronbach’s alpha coefficients for cognitive abilities, experience, and intuition were 0.95, 0.84, and 0.87 in ED nurses and 0.91, 0.67, and 0.82 in EMTs, respectively.

After obtaining permission from the toolmaker, the questionnaire was translated into Persian by a translator. The validity of the instrument, in terms of content and form, was examined by a group of 12 university professors, and their opinions were considered in the research. The reliability (internal consistency) of the instrument was calculated by calculating Cronbach’s alpha (α = 0.97). The test-retest method was also were used to assess the reliability of the questionnaire. Twenty questionnaires were distributed among hospital and pre-hospital emergency nurses at the same interval, following which the agreement of the answers was evaluated, and a retest coefficient of 0.94 was obtained, which indicated the compatibility of the questionnaire.

### Data Analysis

Data analysis was performed using Social Science Statistical Package (SPSS) version 22. Descriptive analysis was used to describe the characteristics of the samples. One-way ANOVA and t-test were used to determine statistically significant differences between the groups related to specific variables. Factors affecting TDM were identified as predictors via multiple linear regression analysis.

## Result

### General specifications of ED nurses and EMTs and their relationship with the total score of triage decision-making

The overall response rate of this study was 57.4% (472.821). The study sample consisted of 472 participants (mean age: 32.50 ± 6.06). Of the participants, 67.7% (n: 320) were ED nurses and 32.2% (n: 152) were EMTs. The results showed that demographic variables, work experience, and triage training course had a significantly positive relationship with the TDM of ED nurses (P < .05), and age, work experience, and triage training course had a significantly positive relationship with the TDM of EMTs (P < .001) (Table 1).

**Table 1:** Descriptive statistics of participants and their relationship with the total score of TDM in ED nurses (n = 320) and EMTs (n = 152)

Variables	ED Nurses						EMTs					
	Mean	SD	N	%	TDMI Mean	P value	Mean	SD	N	%	TDMI Mean	P value
Age ^a^	32.47	5.67			116.15	r = .073 p = .193	32.55	6.83			121.00	r = 263^**^ p = .001
Gender ^b^ Male female			101 219	34.7 65.3	118.41 115.11	t = 0.302 p = .194			146 6	96.1 3.9	121.11 118.33	u = 0.142 * p = .887
Marital Status ^b^ Single Married			111 209	34.1 65.9	115.32 116.59	t = − 0.513 p = .608			52 100	34.2 65.8	120.38 121.33	t =-0.357 p = .721
Work experience ^a^	7.98	5.04			116.15	r = .130* p = .020	8.25	6.56			121.00	r = .287^**^ p = .000
work shift ^b^ Fixed work shift rotating shifts			44 276	13.8 86.3	118.43 115.79	t = 0.770 p = .442			15 137	9.9 90.1	120.60 121.05	t = − 0.107 p = .915
Level of Educational ^c^ Associate Bachelor Master or PhD			5 295 20	1.6 92.2 6.2	118.40 116.07 116.80	F = 0.057 p = .811			43 106 3	28.3 69.7 2.0	120.53 121.29 117.66	F = 0.107 p = .898
Triage training course ^b^ YES NO			253 67	79.1 20.9	117.82 109.86	t = 2.773 p = .006			130 22	85.5 14.5	122.30 113.31	t = 2.576 p = .011
Service location ^b^ Center of province countryside			222 98	69.4 30.6	115.95 116.61	t = − 0.256 p = .798			105 47	69.1 30.9	119.44 124.48	t =-1.877 p = .062

*u = Mann-Whitney U a = Correlation test b = T-test c = ANOVA

## Descriptive statistics of triage decision-making and its dimensions

Statistical analysis showed that there was a significant statistical relationship between the triage decision score in ED and EMTs (Table 2).

**Table 2:** Descriptive statistics the study variables in ED nurses (n = 320) and EMTs (n = 152)

	Skills	Mean( SD)	Minimum	Maximum
**ED Nurses**	Cognitive abilities	62.60 ± 11.912	14	84
	Experience	25.62 ± 5.364	6	36
	Intuition	27.92 ± 6.620	7	42
	Total TDMI	116.15 ± 21.102	27	162
**EMTs**	Cognitive abilities	66.51 ± 9.525	40	84
	Experience	26.59 ± 3.973	16	36
	Intuition	27.89 ± 6.095	10	41
	Total TDMI	121.00 ± 15.428	82	158

## Descriptive statistics of triage decision-making based on self-reported levels of nursing proficiency

Based on self-reported levels of nursing proficiency, the highest and lowest levels of TDM in both ED and EMTs were reported for the expert nurses (122.11 ± 19.513) and (124.86 ± 16.749) and novice nurses (109.93 ± 15.863) and (115.11 ± 11.623) respectively. The results showed that self-reported levels of nursing proficiency had a statistically significant relationship with the TDM of ED nurses (Table 3).

**Table 3:** Mean Scores of TDMI and TDMI Subscales in ED nurses and EMTs in Relation to self-reported levels of nursing proficiency

	self-reported levels of nursing proficiency		***Cognitive abilities(SD)***	***Experience(SD)***	***Intuition(SD)***	Total TDMI ± SD
**ED Nurses** (n = 320)	*Novice and* *Advanced beginner*	n = 76	60.36 (12.022)	24.46(5.683)	26.23(6.347)	109.93 ± 15.863 111.06 ± 20.695
	*Competent*	n = 93	61.36(12.0927)	25.73(5.109)	27.45(6.397)	114.54 ± 20.783
	*Proficient*	n = 82	63.41(11.945)	25.78(5.279)	28.48(6.762)	117.68 ± 22.068
	*Expert*	n = 69	65.75(10.953)	26.59(5.320)	29.76(6.635)	122.11 ± 19.513
	F = 3.725 p = .012					
**EMTs** (n = 152)	Novice and Advanced beginner*	n = 51	66.82(9.729)	26.03(3.918)	26.66(6.574)	119.52 ± 15.893
	*Competent*	n = 43	64.90(8.698)	27.04(3.884)	28.41(5.872)	120.37 ± 14.803
	*Proficient*	n = 29	66.89(9.839)	26.17(4.192)	27.62(5.440)	120.68 ± 14.258
	*Expert*	n = 29	67.96(10.171)	27.34(3.984)	29.55(5.979)	124.86 ± 16.749
	F = 0.784 p = .504					

*Due to the small number of ***Novice*** ED nurses and EMTs, it was combined with ***advanced beginner*** in the table

## Predictors of triage decision-making

The results of multiple regression analysis showing the predictors of triage decision-making are shown in Table 4. A multivariate regression analysis was performed using triage decision-making as the dependent variable and general characteristics and self-reported nursing skill levels as independent variables in two groups of emergency nurses. Of these eight variables, five variables in the ED group accounted for 10% (F = 4.314, p < .001) and five variables in the EMTs group accounted for 22% (F = 5.140, p < .001) of the variance of the final model. (Table 4).

**Table 4:** Multiple linear regression predicting Triage decision-making in ED nurses and EMTs

***Variables***	***ED nurses***					***EMTs***				
	B	Std.Error	Beta	T	Sig	B	Std.Error	Beta	T	Sig
*self-reported levels of nursing proficiency*	3.981	1.160	0.219	3.433	0.001	6.450	1.788	0.503	3.608	0.000
*Age*	1.482	0.508	0.399	2.918	0.004	0.252	0.524	0.112	0.482	0.631
*Level of Education*	-2.164	3.789	− 0.031	− 0.571	0.568	0.810	2.576	0.025	0.314	0.754
*Work experience*	1.762	0.562	0.421	3.137	0.002	1.590	0.580	0.676	2.741	0.007
*Marital status*	-5.696	2.623	− 0.131	-2.171	0.031	-6.673	3.095	− 0.206	-2.156	0.033
*Work shift*	-2.970	3.421	− 0.049	− 0.868	0.386	-2.092	4.167	− 0.041	− 0.502	0.616
*Triage Training course*	-9.134	2.841	− 0.176	-3.215	0.001	6.619-	3.293	0.151-	2.010-	0.046
*service location*	0.356	0.642	0.030	0.555	0.579	2.043	0.754	0.205	2.709	0.008
	R square = 0.100 F = 4.314 p = .000					R square = 0.223 F = 5.140 p = .000				

## Discussion

Triage decision making is an essential skill for nurses in all areas of patient care, including ED and EMS settings to categorize patients based on their severity to manage patient flow [13, 20] Triage nurses need to be able to quickly and efficiently determine 20]. In prioritizing a patient’s urgency for an accurate category and triage decision-making, the presence of expert and skillful emergency nurses is essential to determine the best effect of the intervention on the patient’s health [6]. Therefore these study aimed to identify the level of triage decision-making (TDM) and TDM predictors in ED nurses and EMTs based on self-reported levels of nursing proficiency in Benner’s theory from novice to expert.

All decisions, including those made in stressful critical situations, require thorough consideration. We are likely to understand different people’s decisions under the same conditions, and these decisions may influence the whole chain of future studies. Decision making in nursing profession is one of the basic concepts. If timely and correct decisions are made, they can improve the quality of care, accelerate the treatment process, reduce treatment costs, and ensure patient safety [12, 13]. The findings of the study showed that the TDMI total score was higher than the average in both groups of ED nurses and EMTs. The results obtained from this research are similar to the results of the studies conducted by Aktash and Alamdar in 2017 and also by Al-Zahrani and Al-Mutri in 2022 [13, 23]. Triage decision-making is an essential skill for nurses in all areas of patient care, to categorize patients according to their severity to manage patient flow [13, 20]. Triage nurses should be able to make decisions quickly and efficiently [20]. In prioritizing the patient’s urgency for an accurate classification and triage decision, the presence of expert and skilled emergency nurses is necessary to determine the best effect of the intervention on the patient’s health [4, 6]. The quality index of nurses in triage is determined by correct and quick decision-making, deep knowledge and experience [23]. Due to the special conditions of working in the emergency room and the need for a large amount of information in a short time, working in the emergency room requires a high speed of action [24]. Triage decision-making depends on cognitive factors, use of intuition and clinical experience [16]. The personnel working in the emergency room are trained personnel who have extensive knowledge in all fields of medical and nursing sub-groups. Also, interventions and medical services in the emergency environment are based on judgment and independent skills in decision-making and prioritization, which are obtained through training 24]. On the other hand, since the majority of participating EMTs are men due to the fact that Iranian national laws prohibit women from working in pre-hospital emergency, it may have an impact on some study variables and triage decision making. Limitation of human resources is one of the main challenges of EMS [8]. In order to respond to the emergency needs of women in Tehran, Shiraz and Behbahan, special ambulances were launched in 2018, which accommodate two female nurses and one male driver [8].

In both ED and EMTs, the mean scores of the TDM and its subscales based on the self-reported levels of nursing proficiency, from novice to expert, were higher in expert nurses than in novice, advanced beginner, competent, and proficient nurses. This result was consistent with Cone’s findings [22]. Level of expertise influences diagnostic decisions [25]. The decision-making skills of expert nurses are different from those of novice nurses, and this difference is due to experience [15, 26]. Moreover, the results of the study showed that expert EMTs had higher scores in TDM than ED nurses, and this could be because EMTs have a better ability to reflect on past experiences and rely on intuition [4, 10].

The results also showed that the levels of nursing proficiency and work experience were a predictor of TDM in ED nurses and EMTs. Experience and expertise have been reported as the most important factors influencing the decision of triage nurses [3, 27–29]. Studies show that expert nurses in the clinical decision-making process, using macro cognition, use various factors such as new information, environment, and organizational factors. In contrast, novices may use micro cognition, which relies heavily on objective data [30–32]. Experience is a key factor in increasing the likelihood of decision stability [33]. Experts’ decisions may be more accurate than those of novices. Speed of action and rapid decision-making in frontline health care workers are essential to respond appropriately to rapid changes in patient conditions and to correct intervention [4]. The experience facilitates the growth of the nurse’s confidence, allowing the use of subconscious logic to guide decisions [34], and provides many opportunities for nurses to analyze patient management practices throughout the long-term learning process [33]. Regardless of the level of experience of the nurse, there is a need for staff to have triage training before undertaking the role of triage nursing [35]. The lack of trained and expert staff jeopardizes decision-making in the initial encounter with the patient [36].

An appropriate measure to improve the skills and accuracy of nurses in triage decision-making is training [6]. In the present study, the triage training course was identified as the predictor of TDM in ED nurses and EMTs. Several studies have reported that training is the foundation of a triage system and greatly contributes to TDM [12, 28, 29, 37]. The results of this study were in line with those of previous studies [13, 23]. This may indicate the high quality of the courses offered to nurses, the similarity between these courses and the actual working conditions, and the existence of retraining courses for them. Effective triage depends on the qualifications of the triage nurses [38]. Therefore, nursing educators, hospital leaders, and EMS officials should address this important issue and provide triage training well-designed for triage nurses to improve their judgment and decision-making in the future.

The results of the study also showed that marital status is a predictor of TDM in ED nurses. In TDM scores of both ED nurses and EMTs, the singles had relatively low scores than the married ones. Decision-making skills and TDM can be affected by individual and environmental characteristics of nurses [4, 5]. Married people in another environment (family) have high participation in decision-making, and in matters related to life, they are more likely to create alternative methods and choose the best option to solve the problem [39], for this reason, they may be able to make decisions more easily in other environments. Therefore, family attributes can affect an individual’s decision-making skills.

Decision-making skills are affected by the behavioral and individual characteristics of nurses [40]. The results of the study showed age as a predictor of TDM in ED nurses. This was in line with studies that showed that age has a significant relationship with clinical decision-making [15, 40, 41]. With age increase, nurses’ skills and accuracy increase over time, and they make more rational decisions 40]. Older nurses are more mature in their thinking, and the ability to think critically grows with age, allowing the individuals to practice more reasoning in a variety of situations [33]. Age increase provides the conditions for gaining experience. With age increase, individuals can create new alternatives and add them to their previous list of behaviors [39]. Therefore, it will provide better decision-making power in similar cases based on previous experiences.

The results of the study showed that the service location in EMTs was a predictor of TDM, and EMTs who worked in the countryside had higher TDM skills. There are differences in environmental factors related to professional development in large and small cities [38]. Other studies have reported a link between the service location and triage competency and decision-making [38, 42]. However, these results were not consistent with the results of the present research. In the countryside, EMTs face fewer workloads and fewer patients, so they have more time to decide on triage and rush-free triage [38]. Also, with technological advances, educational resources are readily available and used by rural nurses.

## Conclusion

In today’s healthcare systems, patients usually receive their first care from EMTs and ED nurses. The more correctly, accurately, and quickly these professionals act, the lower will be the rate of fatalities and possible disabilities and the more trust people can place in the services provided by ED and EMS. Effective TDM skills can not only help nurses make the right decisions, but also increase their flexibility and enable them to adapt to difficult and unpredictable situations. Therefore, hospital and EMS managers must improve the quality of TDM skills of personnel. In this study, self-reported levels of nursing proficiency, age, work experience, marital status, triage training course, and service location constituted the factors that influence the TDM of EMTs and ED nurses. Understanding the predictors influencing TDM health professionals (ED nurses and EMTs) may facilitate the understanding of their training needs. Given that the training needs of a novice and inexperienced person may be different from those of an expert person, it is recommended that the training methods be based on the experiences and professional levels of nurses so that the training provided is effective and quality. Moreover, to increase the TDM power and reduce TDM errors due to lack of experience, a system is suggested to be established to allow novice nurses in the first year to work with experienced nurses. Also it is suggested that the determining educational and training focus with regards to triage before entering the bedside be done based on predictors.

## Limitation

There are several limitations to consider in this study. First, the findings are from a selected geographical location in Ardabil-Iran within a publicly funded healthcare system. Due to the differences in the cultural and social conditions of Iran from other countries, the results may not be completely generalizable, so similar studies should be conducted in other countries. Second, the present study measured variables using only self-reported questionnaires, which, in turn, can expose the results to bias. Moreover, other assessment tools and methods such as interviews are recommended to be used to acquire more accurate and credible results. Furthermore, considering the special requirements of the nursing profession and fatigue, workload, and time constraints in completing the questionnaires during the outbreak of COVID-19, it is recommended to conduct a similar study after complete control of the disease and recruitment of a suitable sample.

## Data Availability

The datasets used and/or analysed during the current study available from the corresponding author on reasonable request.
